# Adapting existing diabetes risk scores for an Asian population: a risk score for detecting undiagnosed diabetes in the Mongolian population

**DOI:** 10.1186/s12889-015-2298-9

**Published:** 2015-09-22

**Authors:** Otgontuya Dugee, Oyunbileg Janchiv, Pekka Jousilahti, Ariuntuya Sakhiya, Enkhtuya Palam, J. Pekka Nuorti, Markku Peltonen

**Affiliations:** Public Health Institute, Ministry of Health, Ulaanbaatar, Mongolia; Department of Health, National Institute for Health and Welfare (THL), Helsinki, Finland; Department of Epidemiology, School of Health Sciences, University of Tampere, Tampere, Finland

**Keywords:** Type 2 diabetes, Risk scores, Undiagnosed, Mongolia, Screening

## Abstract

**Background:**

Most of the commonly used diabetes mellitus screening tools and risk scores have been developed with American or European populations in mind. Their applicability, therefore, to low and middle-income countries remains unquantified. Simultaneously, low and middle-income countries including Mongolia are currently witnessing rising diabetes prevalence. This research aims to develop and validate a diabetes risk score for the screening of undiagnosed type 2 diabetes mellitus in the Mongolian adult population.

**Methods:**

Blood glucose measurements from 1018 Mongolians, as well as information on demography and risk factors prevalence was drawn from 2009 STEPS data. Existing risk scores were applied, measuring sensitivity using area under ROC-curves. Logistic regression models were used to identify additional independent predictors for undiagnosed diabetes. Finally, a new risk score was developed and Hosmer-Lemeshow tests were used to evaluate the agreement between the observed and predicted prevalence.

**Results:**

The performance of existing risk scores to identify undiagnosed diabetes was moderate; with the area under ROC curves between 61–64 %. In addition to well-established risk factors, three new independent predictors for undiagnosed diabetes were identified. Incorporating these into a new risk score, the area under ROC curves increased to 77 % (95 % CI 71 %–82 %).

**Conclusions:**

Existing European or American diabetes risk tools cannot be adopted in Asian countries without prior validation in the specific population. With this in mind, a low-cost, reliable screening tool for undiagnosed diabetes was developed and internally validated for Mongolians. The potential for cost and morbidity savings could be significant.

## Background

Type 2 diabetes (T2D) is a common disease, and it’s prevalence has been increasing around the world [1]. Around half of all individuals with T2D are undiagnosed [2, 3]. The disease is characterized by a long asymptomatic preclinical stage with disturbances in glucose metabolism, such as impaired fasting glucose (IFG) and impaired glucose tolerance (IGT). Further, these stages are often associated with the metabolic syndrome as well as other risk factors for vascular diseases, and they are associated with development of micro- and macrovascular complications in the course of the disease [4, 5]. Sometimes even before the clinical diagnosis of diabetes [6]. Thus, undiagnosed diabetes and disturbances in glucose metabolism in general are associated with increased risk of death, by as much three times [7–9].

Early detection of T2D is likely to be beneficial from the point of view of the individual, as early management of the disease, its related comorbidities and their risk factors might lead to benefits such as reduced morbidities and improved quality of life [10, 11-13]. These benefits might in turn translate to benefits to the society at large, in terms of reduced social and economic burden [14, 15].

Several T2D risk scores have been developed worldwide in order to improve early detection. These risk scores have been developed in both the prospective setting to identify people who are at increased risk of developing diabetes in the future [16–20], as well as in the cross-sectional setting to identify people with undiagnosed diabetes [21–27, 28–30]. However, before adopting existing risk scores as screening tools in different populations and in different ethnic groups, their performance should be evaluated and validated in the local setting [31].

Mongolia is a central Asian country, bordered by the Russian Federation to the north and the People’s Republic of China to the south. It had a total population of about 2.9 million in 2010, of which some 62 % lived in the urban area mainly in capital city Ulaanbaatar whereas the rest resides in the large rural territory [32]. The life-expectancy at birth was 63.2 years for men and 71.2 years for women in 2011. In a population-based health survey in 1999, the total prevalence of type 2 diabetes was around 3 % when standardized with the global population [33]. Of all persons with diabetes in the survey, approximately two-thirds were unaware of their condition, and one-half of those with previously diagnosed diabetes were not treated for the condition in any way [34, 33].

In this study, we developed and validated a modified risk score for screening of undiagnosed T2D in the cross-sectional setting, specifically for the Mongolian population.

## Methods

### Study population

National representative, cross-sectional survey on the Prevalence of Noncommunicable Disease Risk Factors was conducted in Mongolia in 2009 using a WHO STEPwise approach to chronic disease risk factor surveillance. Details of the study protocol have been published elsewhere [35]. A total of 5438 randomly selected individuals aged 15–64 year-old in both sexes were recruited in the survey. The survey collected information on the risk factors through questionnaire interview, physical measurement and laboratory analysis. According to the study protocol every third person aged 25–64 was selected randomly for the laboratory analysis part of the survey. The sub study was completed with 1470 individuals (out of 1812 individuals assigned) aged 25–64 who had complete information on capillary fasting glucose.

The present analysis included information of the individuals (*N* = 1027) in the age group 35–64 years. The analysis excluded 4 subjects reported to have taken medication in the morning of the study. So, the analysis was conducted in 1023 subjects, however the number further minimized in 1018 with missing values of essential predictor variables. Thus, the total sample size in the present analyses is 627 women and 391 men.

### Anthropometric measurements

Body weight, height, and waist circumference were measured in all survey participants. Body weight was measured in kilograms with electronic scales “GIMA”, which is a bioimpedance device capable measuring body weight, body fat percent, and water, muscle and bone mass. Body height was measured in centimeters using “Somatometre-Stanley 04-116” device, which has the capacity to measure height up to 2 meters with a precision of a millimeter difference. Body mass index (BMI) was calculated.

Waist circumference was measured with “GIMA waist meter”, a non-stretchy tape with precision of one millimeter. Waist circumference was measured by placing the tape around bare abdomen just above upper hip bone.

### Measurement of glucose and definition of diabetes

Concentrations of glucose were measured in peripheral (capillary) blood with dry chemical reagent strips using Accutrend GCT (Glucose, Cholesterol, Triglycerides) equipment. The required fasting time was 12 hours. Individuals with blood glucose levels ≥6.1 mmol/l who were not already on medication for diabetes were defined as having undiagnosed type 2 diabetes (T2D) [36].

### Measurement of blood pressure

Blood pressure was measured three times on the right arm of the survey participant in sitting position using OMRON Model M5 automatic blood pressure monitor. Mean of three measurements was taken for analysis of blood pressure. Hypertension was defined as systolic blood pressure ≥140 mmHg, or diastolic blood pressure ≥90 mmHg, or on medication for high blood pressure [35].

### Assessment of lifestyle factors and medication by questionnaires

Information on medication, history of elevated glucose, smoking habits, physical activity and nutrition were collected with detailed questionnaires, which the study participants answered in a survey interview conducted by trained interviewers.

History of elevated glucose was assigned if a participant’s blood glucose had been measured in the past and a healthcare worker had informed the participant of an elevated finding. In addition, those reporting that they were currently on either insulin therapy or medication for diabetes, or both were defined as individuals with diagnosed, drug-treated diabetes and they were excluded from the present analyses.

Individuals were categorized as taking medication for high blood pressure if they stated that a health care worker had measured their blood pressure and that they were informed as having elevated blood pressure and they had taken medication for high blood pressure during the past two weeks.

Information about both leisure time physical activity and employment-related physical activity was collected. Those who responded that they did vigorous or moderate-intensity sports, fitness or recreational activities which resulted in increases in breathing or heart rate lasting for at least 10 minutes continuously on at least 5 days per week, were categorized as leisure time physically active. Similarly, respondents were categorized as being physically active at work if their employment involved vigorous or moderate-intensity activities causing increases in breathing or heart rate lasting for at least 10 minutes [35]. Sedentary behavior and smoking status were also assessed through questioning.

### Existing risk scores

Two existing diabetes risk scores were applicable to the Mongolian STEPs-survey data and thus to be validated; the Finnish Diabetes Risk Score FINDRISC [19], and the Rotterdam Risk Score [26]. The risk scores have proven internal and external validity [37] and acquire similar characteristics of risk factors with the existing survey participants in Mongolia.

### Ethics

Ethical approval for the survey was obtained from the Medical Ethical Committee in the Ministry of Health, Mongolia. Participation in the survey was voluntary. Participants of the survey were informed about the procedures in the survey and consented. Results of the laboratory analyses were informed to the respondent at the field directly.

### Statistical methods

Demographics and risk factor levels for individuals with and without undiagnosed diabetes are presented as means and standard deviations, or as proportions. Comparisons between the groups are done with unpaired t-tests for continuous variables, and Fisher’s exact test for proportions. For the existing risk scores, the validation was ensured using sensitivity analysis based on area under ROC-curves. This measures the discriminatory ability of the scores.

Logistic regression models were used to identify additional independent predictors for undiagnosed diabetes. The final model was reached by fitting a model of all variables significant in univariate analysis and refitting a model with only the variables significant in the multivariate model. In addition to the area under ROC-curves, calibration plots with corresponding Hosmer-Lemeshow tests for goodness of fit were used to evaluate the agreement between observed and predicted prevalences. The coefficients of the final prediction model were transformed to scores by scaling and rounding the coefficients so that the totals score points would be 22.

As we did not have an external sample which could be used for validation of the developed score, an internal validation utilizing bootstrap sampling was conducted [38]. From the original study population, 1000 random samples with replacement were drawn, and the model development process was repeated in each of them. The resulting 1000 prediction models from these bootstrap samples were then evaluated with the original study sample data regarding discrimination and calibration. This validation gives an estimate of possible overfitting in the model development process. All analyses were conducted with statistical software Stata v.10.1.

## Results

Among the 1018 individuals with complete data on all relevant variables, 59 (5.8 %, 35 men and 24 women) were identified as having undiagnosed diabetes. In general, they had worse risk factor profile as compared to the individuals without diabetes, with exception for age (Table 1). Measurements on body weight, waist circumference and blood pressure, as well as factors related to physical activity, nutrition, medication for hypertension and history of known elevation in glucose levels were more common among those with undiagnosed diabetes.

**Table 1:** Characteristics of participants with blood glucose measurement in the Mongolian STEPS 2009 survey. Men and women aged 35-64 years by diabetes status

	Without diabetes (*N* = 959)		Undiagnosed diabetes (*N* = 59)				Total population		
	Mean / %	SD	Mean / %	SD	Difference	p	Mean / %	SD	n
Sex, men, %	37.1		59.3		−22.2	0.001	38.4		1018
Age, years	46.3	8.1	47.6	7.7	−1.3	0.222	46.4	8.1	1018
Body weight, kg	68.6	13.2	75.9	14.9	−7.3	<0.001	69.0	13.4	1018
Body height, kg	160.0	7.9	163.1	8.9	−3.1	0.004	160.2	8.0	1018
BMI, kg/m^2^	26.8	4.7	28.5	4.8	−1.7	0.007	26.9	4.7	1018
Waist circumference, cm	88.8	12.6	95.3	12.2	−6.5	<0.001	89.2	12.7	1018
Systolic blood pressure, mmHg	133.9	23.1	144.1	25.6	−10.2	0.001	134.5	23.3	1018
Diastolic blood pressure, mmHg	85.4	13.6	92.0	13.1	−6.6	<0.001	85.8	13.6	1018
Medication for hypertension, % yes	17.1		30.5		−13.4	0.014	17.9		1018
Hypertension or medication, % yes	44.5		71.2		−26.7	<0.001	46.1		1018
History of elevated glucose, % yes	3.5		10.2		−6.6	0.024	3.9		1018
Leisure time phys. act. daily, % no	60.3		79.7		−19.4	0.003	61.4		1018
Phys. act. at work daily, % no	33.5		35.6		−2.1	0.777	33.6		1018
Sitting time/day, hours	2.9	1.9	4.0	2.8	−1.1	<0.001	2.9	2.0	1018
Sitting time 6h or more/day, % yes	16.0		35.6		−19.6	<0.001	17.1		1018
Fruits weekly, % no	54.8		67.8		−12.9	0.892	55.6		1018
Vegetables weekly, % no	33.2		18.6		14.5	0.351	32.3		1018
Daily smoking, % yes	23.0		33.9		−10.9	0.081	23.7		1018
FINDRISC score (coefficients)^a^	3.9	6.3	8.0	14.4	−4.1	<0.001	4.1	7.1	1018
FINDRISC score (score points)^b^	5.8	3.6	7.4	4.0	−1.6	0.001	5.9	3.6	1018
Rotterdam score^c^	11.7	5.5	14.7	6.8	−2.9	<0.001	11.9	5.7	1018

^a^FINDRISC score: based on the original coefficients in the model. Variables included age, BMI, waist circumference, use of antihypertensive medication, history of high blood glucose, physical activity, and daily consumption of vegetables, fruits or berries

^b^FINDRISC score: based on the transformed coefficients expressed as score points

^c^Rotterdam score: Variables included age, sex, use of antihypertensive medication and presence of obesity

Reflecting the more unfavorable risk factor distribution among those with undiagnosed diabetes, both of the existing risk scores, the FINDRISC and the Rotterdam risk score, were higher among those with undiagnosed diabetes (Table 1). However, the discriminatory power of these scores was limited. The areas under the curves were 61.0 (95 % CI: 54.7-68.3) for the FINDRISC, and 63.9 (95 % CI: 56.4-71.3) for the Rotterdam score.

In univariate logistic regression analyses, most of the variables presented in Table 1 were statistically significantly associated with undiagnosed diabetes (Table 2). In multivariate logistic regression, sex, waist circumference, hypertension or medication for high blood pressure, history of elevated glucose, leisure time physical activity and sitting time 6 hours or more during day were all independently associated with undiagnosed diabetes (Table 2). The area under ROC curve for this model was 76.1 (95 % CI 70.1-82.1), indicating marked improvement over the existing risk scores’ performance.

**Table 2:** Logistic regression models on prevalent undiagnosed type 2 diabetes in the Mongolian STEPS 2009 survey. 1018 men and women aged 35-64 years, of whom 59 had undiagnosed diabetes. Regression coefficients, standard errors (SE) and odds ratios (OR) with 95 % confidence intervals (CI) are given

	Univariate model						Multivariate model			
	β coefficient	SE	Odds ratio	95 % CI	p	β coefficient	SE	Odds ratio	95 % CI	p
Sex (men/women)	0.90	0.27	2.47	(1.45–4.22)	0.001	0.74	0.28	2.10	(1.21–3.66)	0.009
Age, years	0.02	0.02	1.02	(0.99–1.05)	0.188	-	-	-	-	-
Body weight, kg	0.04	0.01	1.04	(1.02–1.05)	0.000	-	-	-	-	-
Height, kg	0.05	0.02	1.05	(1.01–1.09)	0.007	-	-	-	-	-
BMI, kg/m^2^	0.07	0.02	1.07	(1.02–1.12)	0.004	-	-	-	-	-
Waist circumference, cm	0.04	0.01	1.04	(1.02–1.06)	0.000	0.02	0.01	1.02	(1.00–1.05)	0.016
Systolic blood pressure, mmHg	0.02	0.00	1.02	(1.01–1.03)	0.001	-	-	-	-	-
Diastolic blood pressure, mmHg	0.03	0.01	1.03	(1.02–1.05)	0.000	-	-	-	-	-
Medication for hypertension (yes/no)	0.76	0.30	2.13	(1.19–3.80)	0.011	-	-	-	-	-
Hypertension or medication (yes/no)	1.12	0.29	3.08	(1.73–5.49)	0.000	0.88	0.32	2.42	(1.30–4.50)	0.005
History of elevated glucose (yes/no)	1.12	0.47	3.08	(1.24–7.66)	0.016	1.14	0.47	3.13	(1.26–7.81)	0.014
Leisure time phys. act. daily (no/yes)	0.95	0.33	2.58	(1.35–4.93)	0.004	0.79	0.34	2.20	(1.14–4.24)	0.019
Phys. act. at work daily (no/yes)	0.09	0.28	1.10	(0.63–1.90)	0.738	-	-	-	-	-
Sitting time/day, hours	0.21	0.05	1.23	(1.11–1.37)	0.000	-	-	-	-	-
Sitting time 6h or more/day (yes/no)	1.07	0.29	2.91	(1.66–5.10)	0.000	0.82	0.30	2.27	(1.27–4.07)	0.006
Fruits weekly (no/yes)	−0.06	0.27	0.94	(0.55–1.59)	0.811	-	-	-	-	-
Vegetables weekly (no/yes)	−0.50	0.44	0.61	(0.26–1.43)	0.255	-	-	-	-	-
Daily smoking (yes/no)	0.54	0.29	1.71	(0.98–3.00)	0.060	-	-	-	-	-
Area under ROC curve						76.1				
95 % CI						(70.1-82.1)				

**Fig. 1:**
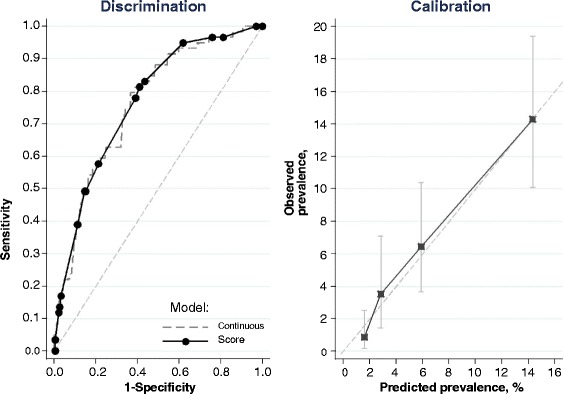
Discrimination and calibration of the final prediction model for prevalence of undiagnosed diabetes in 35-64 years old men and women in the Mongolian STEPS-survey 2009. Discrimination plot (left panel) contains the model with original continuous variables (dashed line) and the final score points (solid line). Calibration plot (right panel) compares the observed and predicted prevalences of diabetes by quartiles of predicted prevalences

Categorizing waist circumference into 2 groups further refined the multivariate prediction model (≥90 cm, and ≥80 cm for men and women, respectively), and the parameters were then re-estimated (Table 3). All the variables in the model were still statistically significant, and the discrimination was 76.2 (95 % CI: 70.2-82.2). Finally, the coefficients of the prediction model were transformed into score points, so that the sum of the scores would maximally be 22. The discrimination with this score system was virtually unchanged. Sensitivities and specificities for the original model with continuous variables and the final score are presented in Fig. 1 (left panel).

**Table 3:** Final logistic regression model on prevalent undiagnosed type 2 diabetes with corresponding scores in the Mongolian STEPS 2009 survey. Regression coefficients, odds ratios and 95 % confidence intervals (CI) are given. 1018 men and women aged 35-64 years, of whom 59 had undiagnosed diabetes

	β coefficient	Odds ratio	95 % CI	Score
Intercept	−5.223			
Sex:				
Women (reference)	-	-		0
Men	0.946	2.58	(1.48–4.49)	4
Waist circumference, cm:				
Men <90/ Women <80 (reference)	-	-		0
Men ≥90/ Women ≥80	0.822	2.28	(1.16–4.46)	3
Hypertension or medication:				
No (reference)	-	-		0
Yes	0.919	2.51	(1.39–4.51)	4
History of elevated glucose:				
No (reference)	-	-		0
Yes	1.108	3.03	(1.17–7.85)	5
Leisure time physical activity daily:				
Yes (reference)	-	-		0
No	0.798	2.22	(1.15–4.28)	3
Sitting time 6h or more/day:				
No (reference)	-	-		0
Yes	0.853	2.35	(1.30–4.25)	3
Score points range				0-22
Area under ROC curve	76.2			76.7
95 % CI	(70.2–82.2)			(70.9–82.4)

Hypertension or medication: Systolic blood pressure ≥140 mmHg, or diastolic blood pressure ≥90 mmHg, or on medication for high blood pressure. History of elevated glucose: healthcare worker informed of an elevated glucose finding in the past

Leisure time physical activity daily: Vigorous- or moderate-intensity sports, fitness or recreational activities which resulted in increases in breathing or heart rate lasting for at least 10 minutes continuously on at least 5 days per week. Sitting time 6h or more/day: Based on question “How much time do you usually spend sitting or reclining on a typical day?”

In addition to the discrimination of the prediction model, also the agreement between the predicted prevalences based on the score points, to the actually observed prevalences was good (Fig. 1, right panel). There were no marked deviations from the identity line between observed and predicted prevalences, and the Hosmer-Lemeshow test was non-significant (*p* = 0.44), indicating adequate goodness-of-fit. Further, the agreement between predicted and observed risk was equally good in men and women (Hosmer-Lemeshow test *p* = 0.83 in men and *p* = 0.50 in women).

Sensitivities, specificities and predicted values for the score are presented with different cut-off values in Table 4. The cut-off point 9 on the score was identified as mathematically most optimal in terms of sensitivity and specificity. With this cut-off, 43.4 % of the study population would have been identified as eligible for further diagnostic testing, and 81.4 % of all individuals with undiagnosed diabetes would have been consequently detected. Increasing the cut-off value decreases the proportion of study population to be included in diagnostic testing markedly, and correspondingly specificity and positive predictive values increases. However, this happens at the cost of a decrease in sensitivity.

**Table 4:** Sensitivity, specificity, positive predictive value, and negative predictive value for selected cut-off values of the modified risk score in the Mongolian STEPS 2009 survey. 1018 men and women aged 35-64 years, of whom 59 had undiagnosed diabetes

Risk score cut-off point:	Proportion of sample, %	Sensitivity, %	Specificity, %	PPV, %	NPV, %
≥0	100.0	100.0	0.0	5.8	-
≥ 3	97.2	100.0	2.9	6.0	100.0
≥ 4	82.1	96.6	18.8	6.8	98.9
≥ 5	77.3	96.6	23.9	7.2	99.1
≥ 6	77.1	96.6	24.1	7.3	99.1
≥ 7	63.9	94.9	38.0	8.6	99.2
≥ 8	45.7	83.1	56.6	10.5	98.2
≥ 9	43.4	81.4	58.9	10.9	98.1
≥ 10	41.3	78.0	61.0	11.0	97.8
≥ 11	23.4	57.6	78.7	14.3	96.8
≥ 12	17.1	49.2	84.9	16.7	96.4
≥ 13	16.5	49.2	85.5	17.3	96.5
≥ 14	12.8	39.0	88.8	17.7	95.9
≥ 15	4.0	16.9	96.8	24.4	95.0
≥ 16	3.1	13.6	97.5	25.0	94.8
≥ 17	2.8	11.9	97.7	24.1	94.7
≥ 18	0.7	3.4	99.5	28.6	94.4

Sensitivity: Proportion who fulfill the cut-off criteria among those with undiagnosed diabetes

Specificity: Proportion who do not fulfill the cut-off criteria among those without undiagnosed diabetes

PPV: Positive predictive value: Proportion with undiagnosed diabetes among those who fulfill the cut-off criteria

NPV: Negative predictive value: Proportion without undiagnosed diabetes among those who do not fulfill the cut-off criteria

Cut-off point ≥ 9 most optimal according to the Youden-index

The results from the internal validation with bootstrap samples indicated that the area under ROC curve was reduced to 72.1 % when taking into consideration the uncertainty in the model development process, as compared to the observed 76.3 % in the original sample. Further, the bias in absolute risk prediction for undiagnosed diabetes was 1.7 %-units in the highest risk quartile (i.e. it is expected that in external samples, the risk prediction will be 1.7 %-units higher than what actually will be observed).

## Discussion

In this population-based study with a random sample of the Mongolian adult population, we identified independent predictors for undiagnosed T2D. In addition to the well-established risk factors sex, waist circumference, hypertension, and history of elevated glucose, also leisure time physical activity and total time spent sitting during daytime hours were identified as independent predictors for undiagnosed diabetes mellitus. The discrimination between those with and without undiagnosed diabetes was improved as compared to the performance of existing risk scores. Further, the agreement between predicted prevalences and those actually observed in different categories of predicted risk was good for both men and women. An internal validation with bootstrap sampling indicated that the discrimination and calibration were reasonably good when taking into account possible overfitting in the model development process.

For reporting, a score was chosen rather than regression coefficients, making it an easy tool for counseling practice of health practitioners as well as individuals self-assessment. We identified that most of the established risk factors for T2D, including sex, waist circumference, hypertension, history of elevated glucose, and physical activity, were also independent predictors of undiagnosed T2D in this population. Physical activity was here defined specifically as at leisure time, because physical activity at work was not associated with diabetes. In addition, sedentary behavior was strongly associated with diabetes, and therefore included in the score. In contrast to most other studies, age was not associated with undiagnosed diabetes. This is somewhat surprising as the ages in this study covered a wide range of ages from 35 to 64 years. Further, BMI did not remain statistically significant predictor in the multivariate model which included waist circumference. This was the case also in the development of the Chinese risk score [21], even though in European populations both waist circumference and BMI have been independently associated with diabetes [19].

In the model development process applied in this study, we started with two existing proven risk scores and evaluated their performance in the Mongolian adult population. For both risk scores included in the evaluation, the Finnish FINDRISC score [19] and the Rotterdam study score [26], the validation of discrimination indicated less than adequate performance. There are several possible reasons for this. The characteristics of the study populations may differ so that the observed associations might be different, resulting in poor performance. For example, the mean age of the study population in the Rotterdam study was 67 years, as compared to 46 years in the present study. Second, differences in case definitions might lead to differences in prediction accuracy. In the Finnish study, diabetes was defined as approval for free-of-charge drug treatment for diabetes, and in the Rotterdam study, oral glucose tolerance test was used for classification of diabetes. Finally, the Finnish score was originally developed in a prospective setting [19]. It has since then been validated several times also in cross-sectional setting, but the performance has in general always been lower as compared to prospective analyses [39].

Several other diabetes risk scores have been developed, both in prospective and cross-sectional settings [16–27]. We were not able to evaluate their performance in detail, as most of the existing scores use information regarding family history of diabetes, and this information was not collected in the Mongolian STEP survey. Additionally, information on behavioral risk factors such as diet and physical activity were not included in the most risk scores in Asian settings [21, 28–30]. It is generally accepted that genetic predisposition is important for the development of diabetes [40], and the consistency of information about family history as a factor in several risk scores support that idea. Therefore, we would propose a modification to the risk score presented in this paper with future follow-up surveys.

Several other limitations of this study need to be considered. First, the sample size was relatively small. Therefore, we did not attempt to make prediction models separately for men and women. However, the calibration analysis indicated that the performance of the model was accurate in both sexes. Second, we only had measurements of blood glucose at fasting state. It is likely that using oral glucose tolerance test we would have detected more individuals with early disturbances in glucose metabolism, and this in turn could have resulted in a more sensitive screening tool. Taken together, it is possible that some independent predictors of undiagnosed diabetes were not identified with current analysis; most notably this might be the case for age and BMI. We are hoping that our results could be further validated and possibly updated in coming years with another, independent health survey in Mongolia representing a larger sample.

Our analysis is conducted in a cross-sectional setting, aiming to detect persons with already existing, but undiagnosed diabetes. For optimal primary prevention, identification of people even earlier in the disease-course of diabetes is necessary. With this purpose, the outcome would have been changed to abnormal glucose so that those at increased risk of diabetes in the future according to their glucose levels can be identified as well. However, a prospective study with information on incidence of diabetes would be needed from this specific population in order to validate and develop tools to identify those who are at risk for future onset of diabetes.

## Conclusion

In conclusion, it is evident that through local adaptation and validation of existing European or American-based diabetes risk tools, sensitivity and potential screening efficacy can be increased. This study demonstrates this through the development of a simple, low-cost, yet reliable risk assessment tool for people with undiagnosed diabetes, developed and validated for the Mongolian population. The result is a tool that could now be used for more effective screening within primary care, in a country and region where diabetes is becoming an increasing concern.
